# Forelimb preferences in human beings and other species: multiple models for testing hypotheses on lateralization

**DOI:** 10.3389/fpsyg.2015.00233

**Published:** 2015-03-06

**Authors:** Elisabetta Versace, Giorgio Vallortigara

**Affiliations:** Animal Cognition and Neuroscience Laboratory, Center for Mind/Brain Sciences, University of Trento, Rovereto, Italy

**Keywords:** forelimb asymmetries, lateralization, handedness, vertebrates, invertebrates, evolution, genetic architecture

## Abstract

Functional preferences in the use of right/left forelimbs are not exclusively present in humans but have been widely documented in a variety of vertebrate and invertebrate species. A matter of debate is whether non-human species exhibit a degree and consistency of functional forelimb asymmetries comparable to human handedness. The comparison is made difficult by the variability in hand use in humans and the few comparable studies conducted on other species. In spite of this, interesting continuities appear in functions such as feeding, object manipulation and communicative gestures. Studies on invertebrates show how widespread forelimb preferences are among animals, and the importance of experience for the development of forelimb asymmetries. Vertebrate species have been extensively investigated to clarify the origins of forelimb functional asymmetries: comparative evidence shows that selective pressures for different functions have likely driven the evolution of human handedness. Evidence of a complex genetic architecture of human handedness is in line with the idea of multiple evolutionary origins of this trait.

## FORELIMB PREFERENCES AND HANDEDNESS IN HUMAN BEINGS

Human beings show a clear functional asymmetry in hand use but its role and origins are still debated. Several studies report a consistent preference for the right hand for different tasks in different cultures and societies (e.g., Porac and Coren, 1981; Annett, 1985, 2002; Perelle and Ehrman, 1994; Raymond and Pontier, 2004). This population-level pattern has been referred to as handedness (Marchant and McGrew, 1998), as opposed to right-left hand preferences that fluctuate in time or between individuals and tasks. In this paper we will refer to this definition of handedness, although it must be noticed that a certain degree of context dependency has been noted for motor dominance (Calvert and Bishop, 1998; Leconte and Fagard, 2006). A bias as strong as 90% in favor of right handedness is commonly reported for our species (McManus, 2002).

This scenario has encouraged researchers in investigating the origins and functions of handedness for centuries (Rogers et al., 2013). It has been initially suggested that functional asymmetries might be a human-specific trait (Annett, 1995) but empirical evidence has contradicted this hypothesis. Across more than one hundred non-human vertebrate species, about 70% show limb preferences (Ströckens et al., 2013). Several studies have identified behavioral limb asymmetries in invertebrates too (Frasnelli et al., 2012), although this taxon has been explored to less extent. To summarize a large corpus of studies, asymmetries in limb use have been extensively documented in such a variety of taxa and species that they are clearly not restricted to humans or their proximate relatives. A matter of debate is whether non-human species exhibit a degree and consistency of functional forelimb asymmetries comparable to human handedness. Assuming a 9:1 right-left handedness ratio in humans, other species appear less strongly lateralized (Ströckens et al., 2013), but the pattern of human handedness though is more complex than often reported.

First, several concerns have been raised on the methodology commonly used to assess human handedness (e.g., Marchant et al., 1995; Marchant and McGrew, 1998). In most cases it has been evaluated through self-report questionnaires (Annett, 1970; Oldfield, 1971; Steenhuis et al., 1990). Some questionnaires have demonstrated consistency in time and a good correlation to behavioral measures (Raczkowski et al., 1974; Coren and Porac, 1978) but it should be noticed that the use questionnaires can enhance a response bias. Moreover, these surveys assay cultural-specific activities, such as writing or drawing, and the use of functional objects: throwing a ball, using scissors, a toothbrush, knife and fork, a spoon, a broom, a racket, a shovel, striking a match, opening a box, dealing cards, hammering, unscrew a jar. The exclusion of non-literate societies, and the focus on fine movement and object manipulation can produce an overestimation of the right bias. Moreover, these activities cannot be assessed in non-human animals, thus constraining the possibility of interspecific comparisons. More realistic and comparable evaluations of human handedness should focus on the observation of naturalistic spontaneous behaviors (e.g., Meunier et al., 2012) but few studies have been conducted along this line. Direct observation of spontaneous behaviors in three non-literate societies has revealed an overall frequency of 84% right handedness for tool use, whereas in other actions individuals were mixed-handed and there was an overall right bias of about 55% (Marchant et al., 1995). Evidence for forelimb asymmetries in self-directed behaviors in other primates are less than conclusive but point at a role of emotions and/or difficulty of the task: some studies reported no population-level asymmetries in great apes (Aruguete et al., 1992; Hopkins and de Waal, 1995; Marchant and McGrew, 1996), others a left-hand bias for face touching in orang-utans, gorillas and chimpanzees (Dimond and Harries, 1984) or a tendency to perform more self-directed behaviors with the right hand in more difficult tasks in chimpanzees (Leavens et al., 2001) and a right preference for combined hand and foot responses directed to the body in squirrel monkeys (Aruguete et al., 1992).

Second, considerable evidence shows time and space variability in human handedness—that in some cases has been related to cultural differences (De Agostini et al., 1997; Mandal, 1999; Dahmen and Fagard, 2005; Kushner, 2013)—, as well as inconsistencies between methods used to assess and report it. Stock et al. (2013) and Marchant and McGrew (1998) have reviewed the existent literature concluding that in Western societies the prevalence of reported left-handers varies between 2 and 13%, whereas in other cultures from 2 to 27% (see Faurie et al., 2002 for functional specialization in traditional societies). Similarly, skeletal analysis on thirteen hunter-gatherers populations, Medieval British and 18–19th century British individuals (Stock et al., 2013) has confirmed a large variability in right-biased asymmetry in upper limb morphology. These measures were conducted on the most sensitive regions of the skeleton, that reflect habitual behavior and lateralization. In particular, in the second metacarpal the hunter-gatherers show only 62.5% right bias [a value lower than observed in chimpanzees (Sarringhaus et al., 2005)], whereas the Medieval and the 18–19th century British groups display a right bias higher than 80%. Referring to the remote past, fossil and archeological records show a proportion of 8–20% left-handers for *Homo neanderthalensis* (Uomini, 2011).

To summarize, historical records, direct observations, anatomical, fossil and archeological evidence confirm the presence of right handedness in our species. This evidence though shows also temporal and spatial variability in the prevalence and/or assessment of this trait. It is important to take the variability of human handedness into account to compare this trait with forelimb preferences in other species.

## FORELIMB PREFERENCES IN INVERTEBRATES: EVIDENCE IN ARTHROPODS

Mounting evidence (reviewed in Vallortigara et al., 1999; Vallortigara, 2000; Vallortigara and Bisazza, 2002; MacNeilage et al., 2009) indicates that cerebral lateralization for specific capabilities has appeared before the emergence of vertebrates. How ancient are the origins of forelimb asymmetries has not yet been fully clarified, but they have been documented in different invertebrate species. In particular, arthropods exhibit forelimb asymmetries at the anatomical (Palmer, 2009; Daugeron et al., 2011) and functional level (see Ruppert et al., 2004; Frasnelli et al., 2012).

In crustaceans with asymmetric forelimbs, each claw is specialized for determined motor actions and functions such as fight, attracting females (Govind and Blundon, 1985; Oliveira and Custodio, 1998), grip, hold, grasp, pull, cut during feeding (Hartman et al., 1997). Moreover, the strength of the crusher claw in blue crabs is significantly greater than in the other cutter claw (Govind and Blundon, 1985). But in species without macroscopic anatomical differences the evidence for side preferences and specialization is indirect or restricted to few tasks and motor actions. Studying desert locusts, Bell and Niven (2014) have recently detected individual level asymmetries in the forelimb used to reach across a gap. These asymmetries though are context-dependent and cannot be assimilated to handedness.

In field-collected spitting spiders, Ades and Ramires (2002) have found more frequent left leg losses (likely due to predation), and a left population level asymmetry for assessing preys has been found in the laboratory. In this task the bias was larger in the foremost limb. The larger left leg damage observed in many arachnid species (Heuts and Lambrechts, 1999) suggests that this bias can be widespread but little is known on side and degree of asymmetries in different tasks. Limb asymmetries have been documented in octopuses too (Byrne et al., 2006): wild-collected octopuses prefer using the anterior arms to reach for and explore objects and show left–right preferences at the individual level. These promising findings provide evidence for limb-specialization but investigation in different contexts is needed to clarify the generality of the preference.

Studies on invertebrates are important to understand the ontogeny of limb asymmetries and the role of environmental influences (Palmer, 2012). American lobsters display a random pattern of individual asymmetries in their claws, so that half have the large crusher claw on the left side. In a series of studies, (Govind, 1992, 1984) showed how differential claw use by juveniles induces one claw to transform into a specialized crusher claw, whereas insufficient stimulation during a critical developmental window causes no specialization. These findings show how important behavior can be in inducing and orienting morphological, and subsequently functional, asymmetry.

At present, a comparison between forelimb preferences in invertebrates and human handedness is difficult due to the limited number of studies conducted on invertebrate species and lack of data collected in different contexts within single species. This fact, together with the relative simplicity of the invertebrate nervous system, ease of maintenance, ecological and social differences between closely related species, make this taxon a treasure trove to investigate the evolutionary, genetic and developmental basis of forelimb asymmetries.

## FORELIMB PREFERENCES IN VERTEBRATES: DIFFERENT SPECIES FOR TESTING DIFFERENT HYPOTHESES

The interest for the origin of forelimb preferences and human handedness has motivated a large amount of studies on limb preferences in vertebrates (reviewed in Hopkins, 2006; Vallortigara et al., 2011; Rogers et al., 2013; Ströckens et al., 2013). It has been suggested that forelimb preferences might originate from a functional specialization in the use of hands/paws/forelimbs, such as feeding, tool use or communication; derive from other lateralized functions, for instance dealing with social life, emotions and stress; be a side effect of anatomic asymmetries, developmental or genetic constraints; be produced by a combination of the above options.

The choice of which model system choose to test different hypotheses on the origin of forelimb asymmetries is related to the ecology, behavioral habits and phylogeny of the species. Studies focused on non-human primates have a great potential to help tracking the evolutionary history of human handedness by looking at similarities and differences across species closely related to our own. Studies on more phylogenetically distant species might clarify the issue of lineage-specificity of forelimb asymmetries and the role of ecological, social and behavioral traits in determining functional forelimb asymmetries. In the last years researchers have paid more and more attention in assessing forelimb asymmetries in a variety of tasks. These data are crucial to understand the complex scenario of forelimb preferences.

### FEEDING, TOOL-USE, BIMANUAL ACTIONS, AND TASK DEMANDS

Researchers have investigated whether brain hemispheres show a specialization for feeding that can reflect on hand preferences. Andrew (2002) has proposed that lateralization has emerged in the earliest vertebrates, whose mouth was located on the left side of the head, for perceptual and motor control of feeding. In line with this idea, a bias for the use of right organs (e.g., eye, forelimb, hand and jaw used in feeding) controlled by the left brain hemisphere has been identified in all vertebrate classes: fishes (De Santi et al., 2002), amphibians (toads: Bisazza et al., 1996), amphibians (Vallortigara et al., 1998), birds (Friedmann and Davis, 1938; Rogers, 1980; Tommasi and Vallortigara, 1999), mammals (for marsupials see, Giljov et al., 2012), for non-primate mammals (e.g., Clapham et al., 1995), for non-human primates (reviewed in Hopkins, 2006). While investigating forelimb asymmetries in feeding, it has been noticed that in primates simple unimanual behaviors enhance a weaker degree of handedness than bimanual behaviors that require coordination of a supporting and a manipulating hand. This pattern is consistent with the task complexity hypothesis (Fagot and Vauclair, 1991): according to this model, simple tasks as basic unimanual reaching induce weaker lateralized responses than high-level tasks as bimanual coordination. It has been proposed that demanding tasks enhance lateralized preferences, as observed for instance in cats (Wells and Millsopp, 2009). The strong pattern of foot preferences for food holding observed in Australian parrots is consistent with this hypothesis: eight out of nine investigated species exhibited a significant population preference for using the left foot and one species for the right foot, with a strength of lateralization up to 90% (Rogers, 1980). Brown and Magat (2011) documented a correlation between foraging mode and footedness, with birds that extract seeds from seedpods using coordinated foot–beak actions being stronger lateralized than birds feeding on small grass seeds and blossoms, that are normally eaten without using a claw. These data suggest the importance of ecological pressures to shape lateralization in limb use, not specifically to forelimbs.

But the demands in terms of hemispheric specialization might be more important than task’s complexity (Rogers, 2009). Along this line, Forrester et al. (2013) have found that children, chimpanzees (Forrester et al., 2012) and gorillas (Forrester et al., 2011) exhibit stronger right lateralization in response to inanimate objects (non-living functional objects) but not to animate objects (social partner, self). Hence dealing with functional objects would activate the left hemisphere, enhancing a right-hand bias in primates that inherited a left-hemisphere specialization for tool-use (Forrester et al., 2013).

It has been hypothesized that human right-handedness derives from a selective pressure for tool use (Breuer et al., 2005; Greenfield, 2011) or coordinated bimanual actions (Wundram, 1986; Bradshaw and Rogers, 1993; Hopkins et al., 2003) and that has been inherited from an ancestor in common with apes. The continuity for bimanual coordinated actions between Old world monkeys (e.g., Vauclair et al., 2005), great apes (e.g., Hopkins, 1995) and humans (Fagard and Marks, 2000; Jacquet et al., 2012) has a strong empirical support (Meguerditchian et al., 2013 review evidence on baboons, rhesus monkeys, chimpanzees, bonobos, gorillas, human infants). Hopkins (2006) suggested that bimanual coordinated activities may have driven the evolution of human handedness more than the mere tool-use. Variations of the postural and biomechanical factors related to the ecology of the species (i.e., arboreal *vs.* terrestrial species) may constitute another major factor in addition to the complexity of the manual behaviors for explaining the phylogenetic distribution.

Summarizing, empirical evidence suggests that right-handedness in primates is influenced by brain specialization for feeding, tool-use and coordinated bimanual actions, and that it is enhanced by the demands imposed by the task. To date little evidence for presence or absence of limb lateralization in feeding, bimanual actions or tool-use has been reported outside the primate order. This prevents us to test the idea that handedness in tool-use and bimanual coordinated actions has specifically emerged in the primate lineage. Interestingly though, corvids show individual level lateralization for tool-use (Weir et al., 2004) and a population right bias for tool-making (Hunt et al., 2001). Further investigation on marsupials with different postural habits can also shed light into the role of tool use and bimanual coordination in handedness.

### RELATION TO LANGUAGE, GESTURE AND COMMUNICATION

Although both right- and left-handers are to a large extent lateralized for language on the left hemisphere (Ocklenburg et al., 2014b), right-handed individuals are more likely to have the left hemisphere dominant for language than left-handed ones (95 to 70–85%) (Knecht et al., 2000; Perlaki et al., 2013).

For instance Knecht et al. (2000) found an increase of right-hemisphere dominance as a function of the degree of left-handedness. The association between hemispheric dominance for language and handedness has suggested that language and handedness coevolved as human-specific traits (Annett, 1985; Corballis, 2002). Yet, evidence on population-level limb asymmetries in species far related from humans can contradict this uniqueness hypothesis. Population-level asymmetries in limb use have emerged for instance in toads (Bisazza et al., 1996), that prefer to use the right paw to remove materials from their body and to rotate from the upside-down position. This evidence, together with population-level asymmetries detected in other species (Ströckens et al., 2013), shows that owning language is not necessary to show population-level limb preferences.

Interspecific comparisons can help clarifying whether right-handedness evolved together with a left-hemisphere specialization for communicative abilities (Corballis, 2002). Meunier et al. (2012) have used the same test to investigate communicative-associated forelimb preferences in 14–20 month-old human infants and baboons showing for both species a significantly stronger right bias for the communicative task than for grasping. Meguerditchian et al. (2013) have recently reviewed the studies that investigated the involvement of the left hemisphere in gestural communication in human and non-human primates. Right-handedness in our species is associated with communicative gestures such as signing in deaf (Vaid et al., 1989; Grossi et al., 1996), hand movements during conversations (Kimura, 1973), pointing in infants and toddlers (Blake et al., 1994; Jacquet et al., 2012). During development, the connection between communicative gestures and right-handedness is stronger than for non-communicative manual actions (Bates et al., 1986; Cochet and Vauclair, 2010; Jacquet et al., 2012): this evidence suggests a possible independence between communicative and non-communicative actions (see Jacquet et al., 2012; Ocklenburg et al., 2014b). Moreover, an integration between speech and gestural communication has been documented in the activation of different brain areas (Corina et al., 2003; Emmorey et al., 2007; Willems et al., 2007; Andric et al., 2013).

Studying non-human primates can help identifying the existence of left-hemispheric specialization for communication and prerequisites of language. Studies with large samples of non-human primates have consistently reported a pronounced population-level right-handedness for different gestures (see Meguerditchian et al., 2013 for a recent review). Interestingly, the degree of right-handedness was higher for gestural communication than for other actions. Overall, these findings confirm the continuity between humans and other primates in left hemispheric specialization for communication. It has been suggested that this lateralized system, shared by a common ancestor of humans and apes, would be a prerequisite for language dated at least 30–40 million years ago (Meguerditchian and Vauclair, 2006).

### RELATION TO BIPEDAL POSTURE

After MacNeilage (MacNeilage et al., 1987; MacNeilage, 2007) proposed the postural origin theory, several authors (e.g., Westergaard et al., 1998; Corbetta, 2003) have hypothesized a relation between handedness and the acquisition of bipedalism, with upright posture facilitating object manipulation and manual laterality. In agreement with this hypothesis, growing evidence shows an effect of posture (body orientation and postural habit) on manual laterality in non-human primates as well as marsupials, with the presence of stronger lateralization in forelimb use for higher degree of bipedalism.

Upright posture correlates with increased side preference in hand use in many species of prosimians, monkeys and apes (critically reviewed, in Hopkins et al., 2011; Giljov et al., 2012), an effect that could be connected to the increase of manual skills with upright posture. The strength of laterality increases from the strongly quadrupedal mouse lemurs to the more bipedal galagos. In apes, species with higher bipedality such as chimpanzees and bonobos tend to have stronger manual preferences than more quadrupedal species such as gorillas and orang-utans. Gorillas and gibbons, described as more bipedal than orang-utans, are more likely to express population-level lateralization in hand use.

In the last decade the relation between body posture and forelimb preferences has been investigated in lineages other than primates. These studies are important to clarify whether this phenomenon has emerged exclusively in the primate lineage. In analogy with quadrupedal prosimians, two obligatory quadrupedal species, domestic cats (Konerding et al., 2012) and the tree shrews (Joly et al., 2012), are not affected by the postural demands of the task (sitting or standing) in either direction or strength of paw preferences while reaching for food. Due to the variety of postural habits, marsupials are convenient models to study the relation between posture and forelimb preferences. Marsupial quadruped species show no population-level bias in forelimb use (Giljov et al., 2013), contrary to the primary bipedal red-necked wallabies (Giljov et al., 2012). In a mostly bipedal marsupial, the brush-tailed bettong, a left forelimb preference has been documented in all studied unimanual behaviors (Giljov et al., 2012).

Summarizing, in non-human primates and marsupials the between-species pattern indicates that the proportion of lateralized individuals and the strength of forelimb preferences tend to increase with more vertical and bipedal species-typical posture. These findings confirm the hypothesis that species postural characteristics influence manual laterality beyond the order of primates.

## LEFT-HANDEDNESS

Forelimb asymmetries are defined as a relative measure between right and left side. In the previous sections we have seen how right-handedness at least partially reflects the specialization of the left hemisphere for feeding, tool-use, bimanual coordination and communication. In our species, documented (but in some cases weak) differences between left- and right-handed individuals and strong *vs.* weak lateralization include general cognitive ability (Nettle, 2003; Nicholls et al., 2010), personality (Grimshaw and Wilson, 2013), motivation (Brookshire and Casasanto, 2012), perception (Ocklenburg et al., 2010), language (Knecht et al., 2000). Differences between right- and left- handed individuals have been found in non-human primates as well.

Looking at marmosets, Rogers and collaborators (Cameron and Rogers, 1999; Gordon and Rogers, 2010) have shown a connection between handedness and cognitive styles: left-handed marmosets are less explorative in unfamiliar environments and less prompt in showing social facilitation of feeding responses. In rhesus macaques Westergaard et al. (2003) have found that right-handed males, contrary to females, receive more aggressive interactions and less grooming from conspecifics and they are more likely to be submissive (see Howell et al., 2007). To understand forelimb preferences, the connection between left forelimb use and left-hemisphere specialization must be explained.

Several studies show that the activity of the right hemisphere is associated with detecting and responding to unexpected and possibly threatening stimuli, including fear and aggressive responses (Rogers and Andrew, 2002; MacNeilage et al., 2009; Rogers, 2009, 2002). Hence tasks performed by right-handed people with the left hand might reflect the activity of the right hemisphere. Researchers have surveyed large samples of human beings involved in sport activities to look for tasks in which the use of the left hand might be over-represented and found confirming evidence of an overrepresentation of left-handers in interacting sports (Goldstein and Young, 1996; Brooks et al., 2004; Loffing and Hagemann, 2014; Loffing et al., 2014). Based on the proportion of left-handed individuals in interactive sports, Raymond et al. (1996) have proposed that left-handed individuals have a frequency-dependent advantage in fighting. This advantage would maintain a proportion of left-handed individuals in the population as a result of an evolutionarily stable strategy (Ghirlanda and Vallortigara, 2004). Along a similar line, a correlation between the degree of left-handedness and homicide rates has been found, although these data can have several explanations (Faurie and Raymond, 2005).

Left-handedness or reduced right-handedness are linked also to some traits with clinical relevance such as depression (Denny, 2009), schizophrenia (Sommer et al., 2001; Dragovic and Hammond, 2005), higher (but nor pathological) alcohol consumption (Denny, 2011), immune response (Stoyanov et al., 2011), while a reduced right-handedness and inconsistent laterality can accompany specific pathological phenotypes (Carlier et al., 2006; Gérard-Desplanches et al., 2007). Interestingly the association between forelimb asymmetries and modulation of immune response has been found in different species: left-pawded mice show higher phytohemagglutinin- and concanavalin- induced proliferation (Neveu et al., 1988). Abramov et al. (2001) observed that in left-pawded mice thymocytes from the left lobe of thymus have higher concanavalin-stimulated proliferation than those from the right lobe, and right-pawded mice show the reversed pattern. Quaranta et al. (2004) found in left-pawed dogs higher percentage and number of lymphocytes and lower percentages of granulocytes and lower number of γ-globulins compared to right-pawed and ambidextrous dogs. Overall, left hemispheric activation associated to right forelimb preference seems to produce either greater production of stress hormones or greater reactivity to stress that enhances immune response, as confirmed by the negative correlation between right-hand preference and stress reactivity in rhesus macaques (Westergaard et al., 2001). Interestingly, limb preferences are sensitive to experience and can even reflect the tendency toward positive or negative cognitive bias (Rogers, 2010), thus showing the importance of the environment in assessing forelimb asymmetries.

## GENETIC ARCHITECTURE OF FORELIMB PREFERENCES

The heritability pattern of human handedness—e.g., familial history of left-handedness (Annett, 1973; Medland et al., 2010), higher concordance of handedness for monozygotic than dizygotic twins (McManus and Bryden, 1992)—suggests a genetic control of this trait. While reviewing different hypotheses about the origin of handedness and forelimb preferences we have showed that selective pressures for different functions—e.g., tool-use, communication, bipedal posture/task complexity, stress responsiveness—have likely influenced the evolution of forelimb preferences. This multifactorial pattern might reflect a complex genetic architecture of forelimb asymmetries.

The first models of the inheritance of handedness have hypothesized a simple genetic basis of this trait, with a single causative locus with two or more alleles or two loci involved (e.g., Levy and Nagylaki, 1972; Annett, 2002; McManus, 2010). Heritability estimates do not contradict these models (Annett, 1985; Klar, 1996). Such a simple genetic architecture was expected to be identified using traditional molecular approaches or genome-wide association studies. But as reviewed in McManus et al. (2013), to date no associations have been replicated across studies, and in several studies only marginally significant results or no significant associations have been found, even in the presence of large samples (Armour et al., 2014). This pattern of results provides indirect evidence of a complex genetic architecture (Rockman, 2012; Mackay, 2014). Moreover, by screening individuals with pathological conditions several loci have been associated with handedness (Ocklenburg et al., 2014b; reviewed in Brandler and Paracchini, 2014), in particular for dyslexia (Scerri et al., 2011; Brandler et al., 2013) and schizophrenia (Francks et al., 2007), thus providing some support to the polygenic basis of handedness. Genetic linkage studies have also identified different genomic regions connected to handedness [for instance the markers 2p12–q11 (Francks et al., 2002, 2003) and 12q21–23 (Warren et al., 2006)], but little is known about the role of orthologs/homologs in non-human species. In the light of genetic evidence, multi-locus models (McManus et al., 2013) and multi-locus models with partial pleiotropy (Ocklenburg et al., 2014b) have been suggested to explain handedness, but the underlying basis of handedness remains to a large extent elusive.

Understanding the molecular basis of functional asymmetries may help reconstructing the causative relationship between lateralization and specific phenotypes (Bishop, 2013), for instance to understand whether weak laterality is the cause or effect of some neurodevelopmental disorders or the same genetic basis underlies both phenotypes. Given the widespread presence of functional asymmetries in limb use in non-human species (see previous sections), one might wonder why the investigation of the underlying genetics of handedness has not been pursued in model species for which genetic tools are easily accessible such as *Drosophila* or mice. Anatomical and functional asymmetries have been identified in the nervous system of fruit flies (Pascual et al., 2004) and in mice the strength of lateralization has a genetic component (Collins, 1968; Biddle and Eales, 1996, 1999). The murine model species though do not apparently exhibit a population level parallel of human handedness. This consideration should not necessarily constrain the investigation of functional asymmetries in other species, especially considering that even in our species forelimb asymmetries can vary due to cultural, ecological and task/function demands. Since human handedness is correlated with cerebral asymmetry, and genes involved in the development of left–right asymmetry are widespread among different species, left–right asymmetry genes might be considered as candidate genes to investigate more specific domains of lateralization such as forelimb asymmetries. For instance, the marker associated to hand skill and dyslexia rs11855415 (Scerri et al., 2011; Brandler et al., 2013) is connected to the Nodal pathway, which is known to determine the development of left/right asymmetries in a wide range of species (Bamford et al., 2000; Concha et al., 2000; Mercola and Levin, 2001; Grande and Patel, 2009). Interestingly, when expression of this pathway is absent, structural asymmetries in zebrafish are maintained but they are random in direction (Concha et al., 2000). The Nodal pathway can be also involved in the development of human handedness (Brandler and Paracchini, 2014), for which it has been argued that direction and strength of biases may represent independent phenotypes (Ocklenburg et al., 2014a).

It is thus apparent that a convergence of data and theories from different disciplines is necessary to elucidate the origins, mechanisms and evolutionary history of limb preferences. In this regard evidence from other species, even those that lack appendages like limbs but that have asymmetries in brain and behavior, such as zebrafish, may ultimately be crucial for deciphering handedness in humans.

### Conflict of Interest Statement

The authors declare that the research was conducted in the absence of any commercial or financial relationships that could be construed as a potential conflict of interest.
